# Supplementary material for: Exploration of novel αβ-protein folds through de novo design
Source: Nat Struct Mol Biol. 2023 Jul 3;30(8):1132–40. doi: 10.1038/s41594-023-01029-0 (PMC10442233; doi:10.1038/s41594-023-01029-0)
Supplement: Supplementary file 1 — Supplementary Text, Figs. 1–11 and Tables 1–17. [file 41594_2023_1029_MOESM1_ESM.pdf]

nature structural & molecular biology

Article

https://doi.org/10.1038/s41594-023-01029-0

Exploration of novel αβ-protein folds through de novo design

In the format provided by the authors and unedited

Table of Contents

Supplementary Text *2*

Supplementary Figures *7*

Supplementary Fig. 1 | Solved NMR structures. 7

Supplementary Fig. 2 | Continuous and interrupted β-sheets. 8

Supplementary Fig. 3 | 2D ^{1}H-^{15}N HSQC of protein NF1-14. 9

Supplementary Fig. 4 | 2D ^{1}H-^{15}N HSQC of protein NF2-02. 10

Supplementary Fig. 5 | 2D ^{1}H-^{15}N HSQC of protein NF3-03. 11

Supplementary Fig. 6 | 2D ^{1}H-^{15}N HSQC of protein NF4-04. 12

Supplementary Fig. 7 | 2D ^{1}H-^{15}N HSQC of protein NF5-03. 13

Supplementary Fig. 8 | 2D ^{1}H-^{15}N HSQC of protein NF6-02. 14

Supplementary Fig. 9 | 2D ^{1}H-^{15}N HSQC of protein NF7-04. 15

Supplementary Fig. 10 | 2D ^{1}H-^{15}N HSQC of protein NF8-01. 16

Supplementary Fig. 11 | TALOS+ order parameter and the number of NOE distance constraints for each residue. 19

Supplementary Tables *20*

Supplementary Table 1 | Designed sequences of the series of NF1. 20

Supplementary Table 2 | Designed sequences of the series of NF2. 21

Supplementary Table 3 | Designed sequences of the series of NF3. 21

Supplementary Table 4 | Designed sequences of the series of NF4. 22

Supplementary Table 5 | Designed sequences of the series of NF5. 22

Supplementary Table 6 | Designed sequences of the series of NF6. 23

Supplementary Table 7 | Designed sequences of the series of NF7. 23

Supplementary Table 8 | Designed sequences of the series of NF8. 24

Supplementary Table 9 | RMSD between the design model and NMR structure. 25

Supplementary Table 10 | Experimental summary of a series of designs for NF1. 26

Supplementary Table 11 | Experimental summary of a series of designs for NF2. 27

Supplementary Table 12 | Experimental summary of a series of designs for NF3. 27

Supplementary Table 13 | Experimental summary of a series of designs for NF4. 28

Supplementary Table 14 | Experimental summary of a series of designs for NF5. 28

Supplementary Table 15 | Experimental summary of a series of designs for NF6. 29

Supplementary Table 16 | Experimental summary of a series of designs for NF7. 29

Supplementary Table 17 | Experimental summary of a series of designs for NF8. 30

Supplementary Text

NMR structure determination

All αβ-proteins in this study are generally stable and have long lifetime (more than 2~3 weeks) at NMR concentration (0.5~1.0mM), and rarely minor components can be found. On the other hand, there is no noticeable signal change in 2D ^{1}H-^{15}N HSQC at different concentrations (2~10 times dilution) for all samples. Taken together with the results from SEC-MALS analysis, all the samples are considered to be in stable monomeric states throughout the NMR experiments.

Prior to the structure determination, the analyst never has known the designed structures even their sequences (perfectly blind analysis) in order to avoid any arbitrary bias for the automated NMR analysis. Owing to the high sensitivity of modern NMR spectrometers (700~800 MHz equipped with 2nd or 3rd generation of Cryo-probes) and high concentration of samples, significant number of NOE peaks were yielded from NOESY type spectra. More than 80~90% of NOE peaks are assigned by CYANA to obtain well converged structures, supporting high consensus between NOE peaks and calculated NMR structures.

Several CYANA calculations were performed using the ACS (Assigned Chemical Shifts) table, NOE peak table, and dihedral angle constraints to obtain 20 models with the lowest target functions. For the obtained CYANA structures, implicit water refinement calculations were performed by AMBER12 with the ff99SB force field. The dihedral angle constraints and distance constraints including additional chirality and backbone omega angle constraints were converted for the AMBER format using the SANDER tool. In the initial stage of the refinement, energy minimization of 500 steps (250 step: steepest gradient, followed by 250 step: conjugate gradient decent) without electrostatic energy and NMR constraint terms was carried out. A short molecular dynamics calculation (total 30 psec, time step 1.0 fsec, using SHAKE algorism) was followed using electrostatic energy based on the generalized Born model (salt concentration: 0.1 M, disabled Surface Accessibility (SA) function, electrostatic potential radius cutoff: 18 Å) and NMR constraint terms. The temperature was gradually increased from 0 K to 300 K for 1,500 steps, then a dynamic calculation at 300 K for 1,500 steps was performed. In the final stage of the refinement, energy minimization of 2,000 steps was performed with the same energy terms.

The RDC back calculations were used for validating the determined NMR structures. This strategy enhances the reliability of the determined NMR structures, with the geometrical normality (such as Ramachandran plots and vdW clash) and violations for restraints. Although residual dipolar couplings (RDC) can be affected by the local motion of HN-N vectors in wide-range time scales, the effect is not big if a sufficient number (coverage of residues more than 80%) and sufficient amplitude of RDC values (>±10Hz) are obtained. Using the software PALES[1], each model coordinate of a calculated NMR ensemble and a number of experimental RDC values, by means of singular value decomposition (SVD), Saupe matrix can be obtained to estimate Euler angles and amplitudes of alignment tensor. Then using the tensor parameter, PALES can calculate the Pearson's correlation coefficient R_P^{free} between simulated and experimental RDC values. The correlation coefficient greater than 0.9 indicates that the calculated structure can be trustful, unless the RDC values were used for structure calculation as constraints. For severely overlapped residues and residues with an order parameter of less than 0.8 predicted by TALOS+, the RDC values were excluded from the analysis. It would be noteworthy that more than 80% of observed RDC data were used for all of the RDC analyses in this study. Fortunately, the designed αβ-proteins are composed of a few helices and slightly twisting β-sheets, of which orientations are largely different; the RDC analysis using $^{1}D_{1H\text{-}15N}$ is firmly suitable for structure validation.

NF1-14

^{1}H-^{15}N HSQC is shown in Supplementary Fig. 2. The methyl protons of Ile46-Hγ2 and Ile28-Hδ1 are weakly shielded by the aromatic rings of Phe80 and Trp99, respectively. Interestingly, the side-chain of Gln18 is stacked by Tyr38, which is well consistent with the strongly shielded amide proton of the side-chain of Gln18. Unusually down-filed shifted Gln13-Hε2 indicates the formation of a strong hydrogen bond stabilizing the tight packing between the first and third helices. All aromatic rings are nearly the same location when overlaying the designed and NMR structures, which supports both the structures have strikingly the same structure. As shown by 2D ^{1}H-^{15}N HSQC in Supplementary Fig. 2, NF1 does not have any minor components under the NMR condition. In the RDC validation analysis, the NMR structure was slightly better than the designed one (RMS were 0.904 and 0.894, respectively). This strongly supports that the designed and NMR structures are the same in solution.

NF2-02

^{1}H-^{15}N HSQC is shown in Supplementary Fig. 3. This designed protein has two tyrosine residues (Tyr16 and Tyr63). Ile27-Hδ1 and -Hγ2 methyl protons are shielded by the aromatic ring of Tyr16. Gln53-Hε1/2 are slightly shielded, suggesting the ring current effect by Tyr63. As a result of the RDC validation analysis, the correlation coefficient was slightly better than the designed structure (0.923 and 0.916, respectively). Taken together with the shielded protons and RDC scores, the designed protein in solution folds into the designed structure.

NF3-03

^{1}H-^{15}N HSQC is shown in Supplementary Fig. 4. The methyl groups, Ile49-Hγ2 and -Hγ1, Ile71-Hδ1, -Hγ2 and -Hγ1, are shielded by Phe54. Val32 is also close enough to Tyr26 but no shielded proton was found. Arg24-Hβ2/3 is shielded, weakly stacking to Tyr26 (this interaction is not found in the designed structure). The key residues Phe54 and Tyr26 are the same location and orientation, which well explains the shielding effect to the methyl and methylene protons. The RDC validation score of the NMR structure was better than that of the designed structure. As the correlation coefficient was greater than 0.9, the solution structure is trustful and similar to the designed structure.

NF4-04

^{1}H-^{15}N HSQC is shown in Supplementary Fig. 5. Because of the relatively high pH and salt concentration, signals of several residues in hairpins are weak, broad, or missing in the 2D ^{1}H-^{15}N HSQC. Additionally, there are a lot of aliphatic amino acids such as Ile, Val and Leu (total 33 residues) for this protein size; supportive spectra were needed: 3D (H)C(CO)NH and (H)CCH-TOCSY for confirmation of sequential assignment and side-chain assignments, and 3D ^{13}C-HSQC (^{13}C-time domain) –NOESY ^{15}N-HSQC, ^{13}C-HSQC (^{13}C- time domain) NOESY ^{13}C-HSQC for obtaining methyl-methyl NOEs (See Methods in the main text).

A lot of methyl protons are shielded: Ile25-Hδ1 by Tyr13, Ile12-Hγ1 and -Hδ1 by Phe62, Ile80-Hγ2, Leu84-Hδ1/2, and Val95-Hγ1/2 by Phe28, Leu109-Hδ1/2 by Phe31. The side-chain amide signals of Gln88 are very unusual position in 2D ^{1}H-^{15}N HSQC. This is probably because Gln88 is involved in the protein core and forms a hydrogen bond with Leu84-CO. Although the

largest difference is observed for the orientation of Phe31 and Leu106, the overall conformation of the NMR structure is similar to the designed one. The RDC validation scores of the NMR and designed structures were greater than 0.9 (0.917 and 0.918), indicating that their global structures are moderately correct. The side-chain location and orientation in the hydrophobic core except for the above mentioned residues are nearly the same between the designed and NMR structures.

<u>NF5-03</u>

^{1}H-^{15}N HSQC is shown in Supplementary Fig. 6. In this protein, a lot of shielded methyl protons are found in 2D ^{1}H-^{13}C HSQC. Ile46-Hγ2 and -Hδ1, Ile52-Hδ1 and -Hγ2, and Ile60-Hγ2 are shielded by the ring current of Phe64, and Ile25-Hγ2, by Tyr35. Ile24-Hγ2 and Val95-Hγ1/2 are also shielded by the ring current of Phe73. These indicate that the NMR structure has well consensus with the chemical shift data. The location and orientation of aromatic rings in the superimposed design and NMR structures are slightly different, however, the backbone conformations are quite similar. The RDC validation scores for both the designed and NMR structures are greater than 0.90, demonstrating that the solution NMR structure are trustful. Despite the correlation coefficient of the designed structure was slightly better than that of the NMR structure, the shielded methyl protons and location and directions of aromatic rings can be explained more reasonable in the NMR structure.

<u>NF6-02</u>

^{1}H-^{15}N HSQC is shown in Supplementary Fig. 7. Ile15-Hγ2, Leu19-Hδ2 or -Hδ1 are shielded by Tyr4, and Leu71-Hδ1/2 are weakly shielded by Tyr46. Interestingly, the side-chain amide signals of Gln25 are slightly broad and weak, and unusual position in ^{1}H-^{15}N HSQC. This can be explained by the formation of hydrogen bonding to the backbone carbonyl group of Gly51, stabilizing packing between the second helix and the second strand. In the overlaid designed and NMR structures, all of the secondary structures as well as aromatics rings are in very similar positions. The RDC analysis showed that the NMR structure is slightly better than designed structure (0.955 and 0.924, respectively), indicating that the designed structure can be very similar conformation in solution.

NF7-04

^{1}H-^{15}N HSQC is shown in Supplementary Fig. 8. The methyl groups of Leu49-Hδ1/2 are shielded by Tyr92, while Val34-Hγ1/2 seems to be shielded by Phe107. Leu18-Hδ1/2 are shielded by Phe6. Tyr92 shields Leu49-Hδ1/2 as well as Gly1-Hα2/3. Since this protein has more aromatic residues than the other proteins, the wide variety of shielding effects can be used to indicate how correct the NMR structure is. Interestingly, Gln2 and Gln4 stabilize the first strand by stacking their side-chains to the hydrophobic core. Aromatic rings except for Tyr5 have similar locations and orientations in both the designed and NMR structures. RDC errors were near 0.9 for both the designed and NMR structures (0.915 and 0.893, respectively), indicating that the NMR structure is correctly determined and quite similar to the designed structure.

NF8-01

^{1}H-^{15}N HSQC is shown in Supplementary Fig. 9. Methyl protons are shielded by the ring current effect such as Ile39-Hδ1 by Tyr27, Leu17-Hδ1/2 and Leu61-Hδ1/2 by Phe6, Leu7-Hδ1/2 by Tyr36, indicating that the NMR structure is well consistent with the chemical shift data as well as all aromatic tightly packed methyl groups enhancing hydrophobic interaction in the core of protein. Interestingly, the side-chain amide group of Gln65 is nearly involved in the hydrophobic core, as revealed from unusual chemical shifts in 2D ^{1}H-^{15}N HSQC. The side-chain orientations and locations of hydrophobic residues are very similar between the designed and NMR structures. The RDC validation analysis showed very good score for the NMR and designed structures (0.930 and 0.926, respectively), indicating that the designed protein folds in solution as exactly designed.

Supplementary Figures

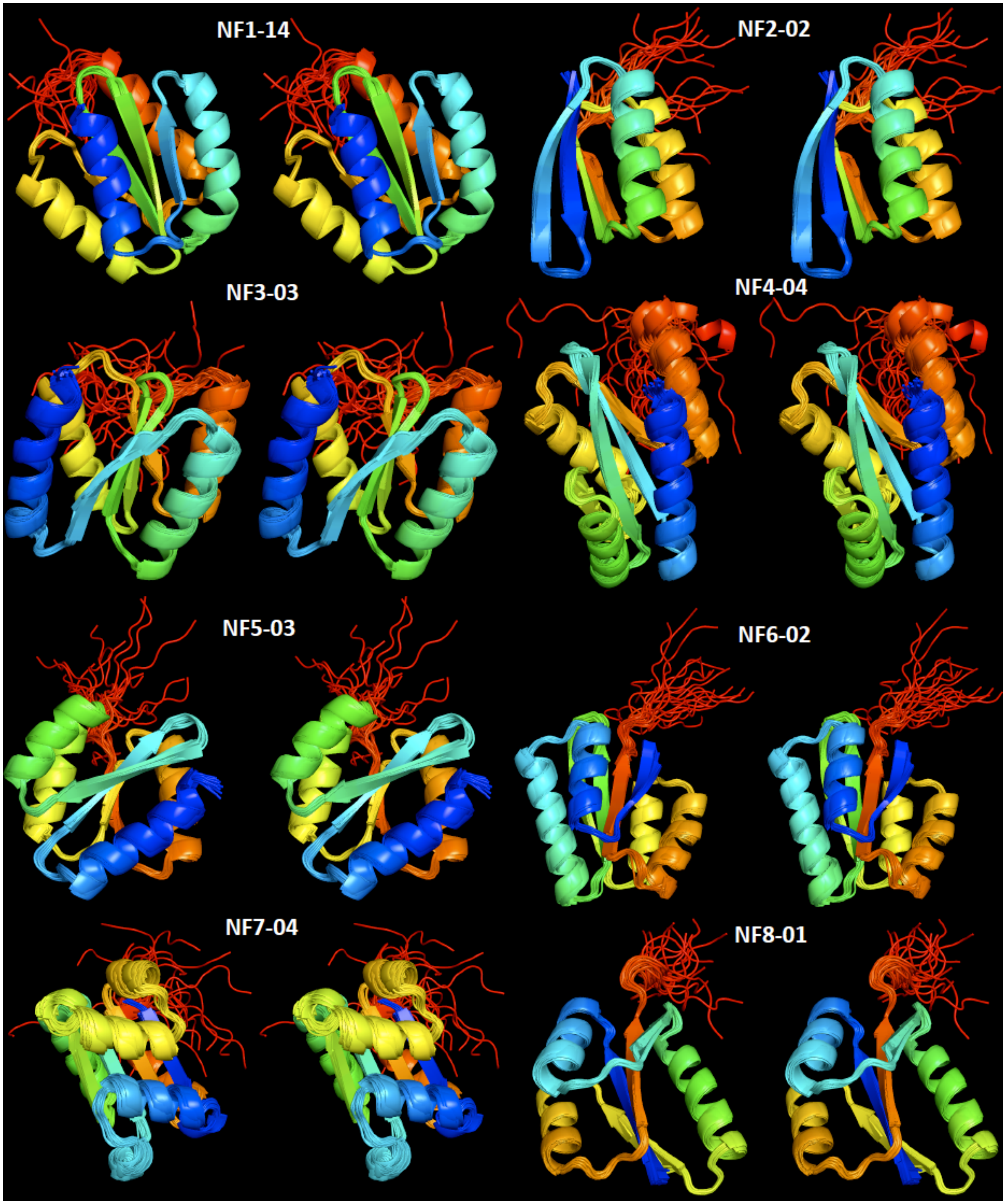

Supplementary Fig. 1 | Solved NMR structures.
Stereo views of superimposed backbone structures of 20 NMR models for each target fold.

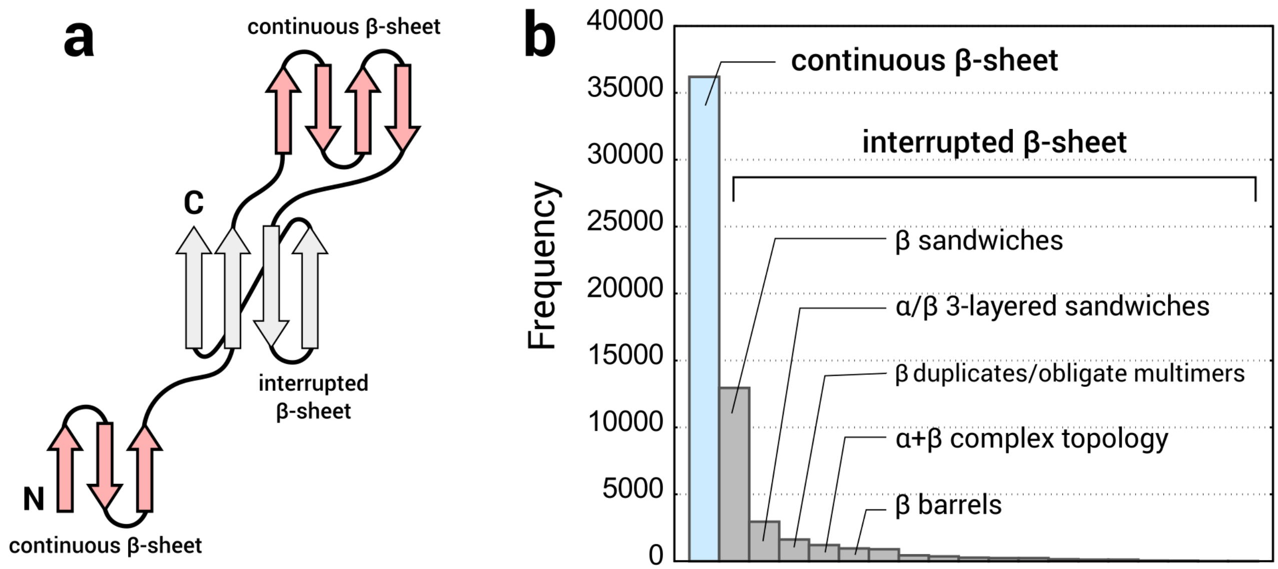

Supplementary Fig. 2 | Continuous and interrupted β-sheets.

a, Illustration of a protein chain comprising three domains, containing a β-sheet each. The red-colored domains have continuous β-sheets without insertion of any other β-sheet domains, whereas the gray-colored domain has an interrupted β-sheet with another β-sheet inserted into the loop region immediately after the first strand. **b**, Observation frequencies for continuous (blue) and interrupted (gray) β-sheets in the ECOD dataset[2]. For interrupted β-sheets, the frequency is shown for each topology pattern (that is, 'Architecture' described in ECOD); the most typical one is the β-sandwich type consisting of two β-sheets facing each other with an entangled chain (e.g. immunoglobulin-like fold).

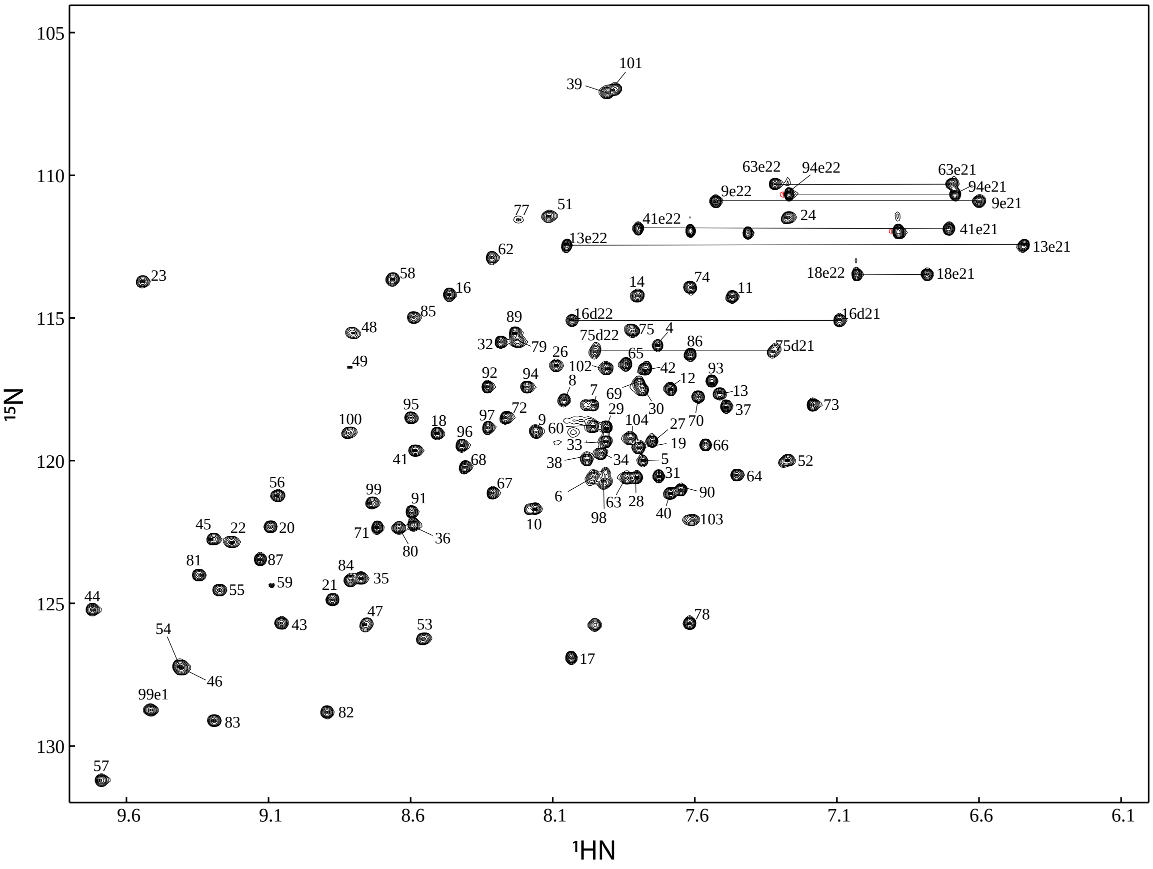

Supplementary Fig. 3 | 2D ^{1}H-^{15}N HSQC of protein NF1-14.

Assigned peaks are labeled with residue numbers. For side-chain amide atoms, the labels are accompanied with d or e or e1, representing Hδ1/Nδ and Hδ2/Nδ for Asn, Hε1/Nε and Hε2/Nε for Gln, and Hε1/Nε1 for Trp, respectively.

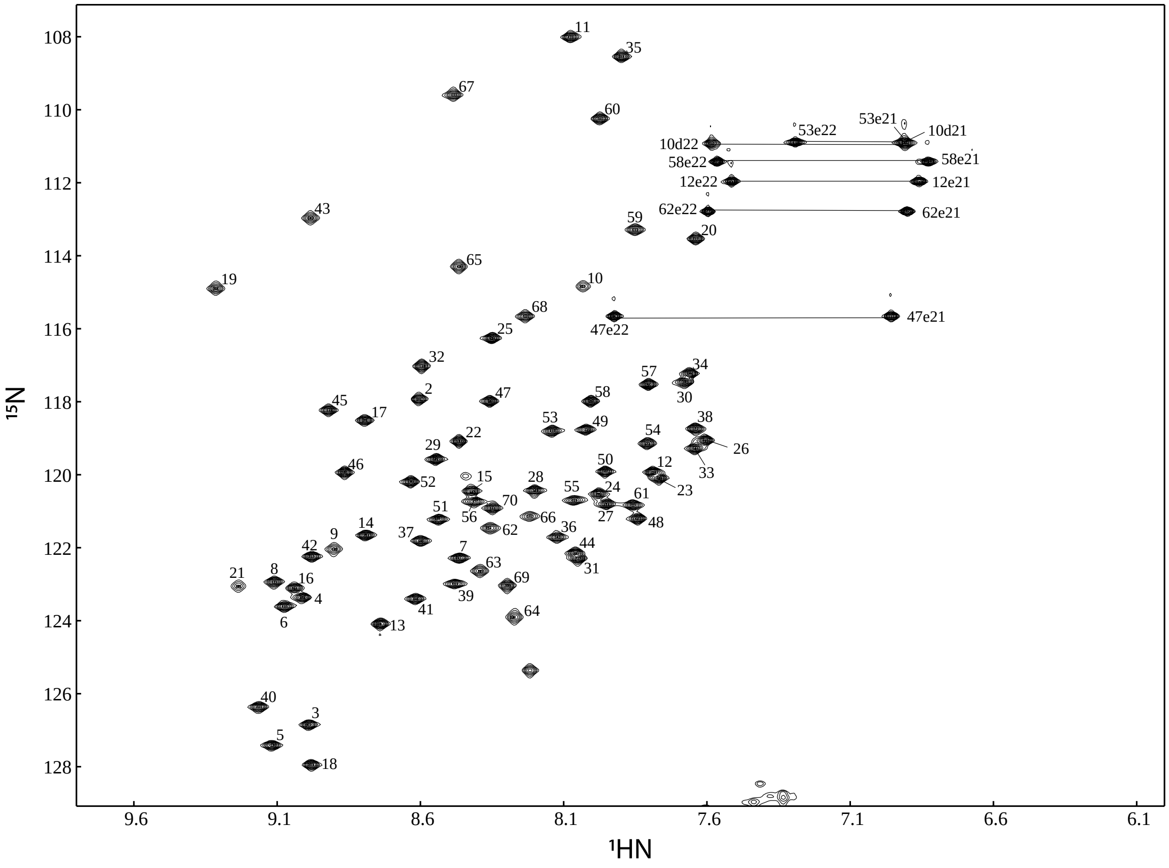

Supplementary Fig. 4 | 2D ^{1}H-^{15}N HSQC of protein NF2-02.

The HSQC spectrum was labeled in the same way as Supplementary Fig. 3.

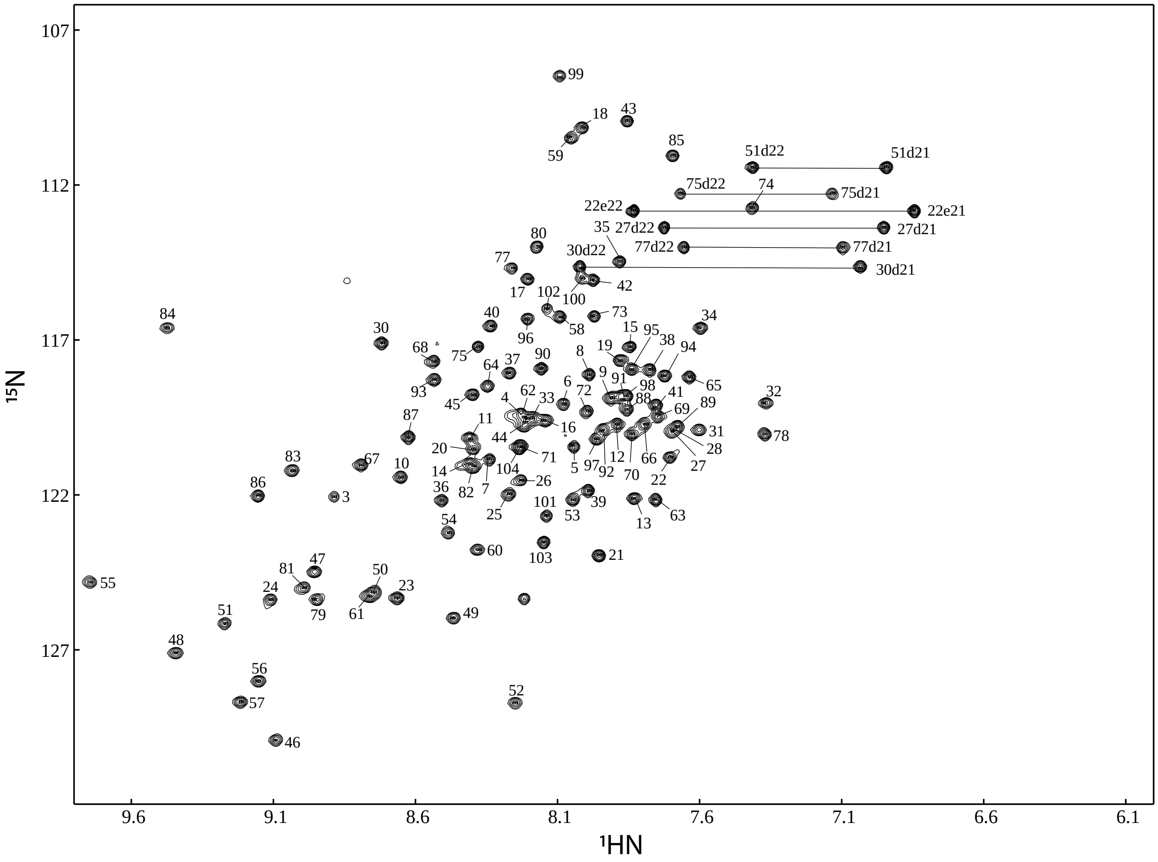

Supplementary Fig. 5 | 2D ^{1}H-^{15}N HSQC of protein NF3-03.

The HSQC spectrum was labeled in the same way as Supplementary Fig. 3.

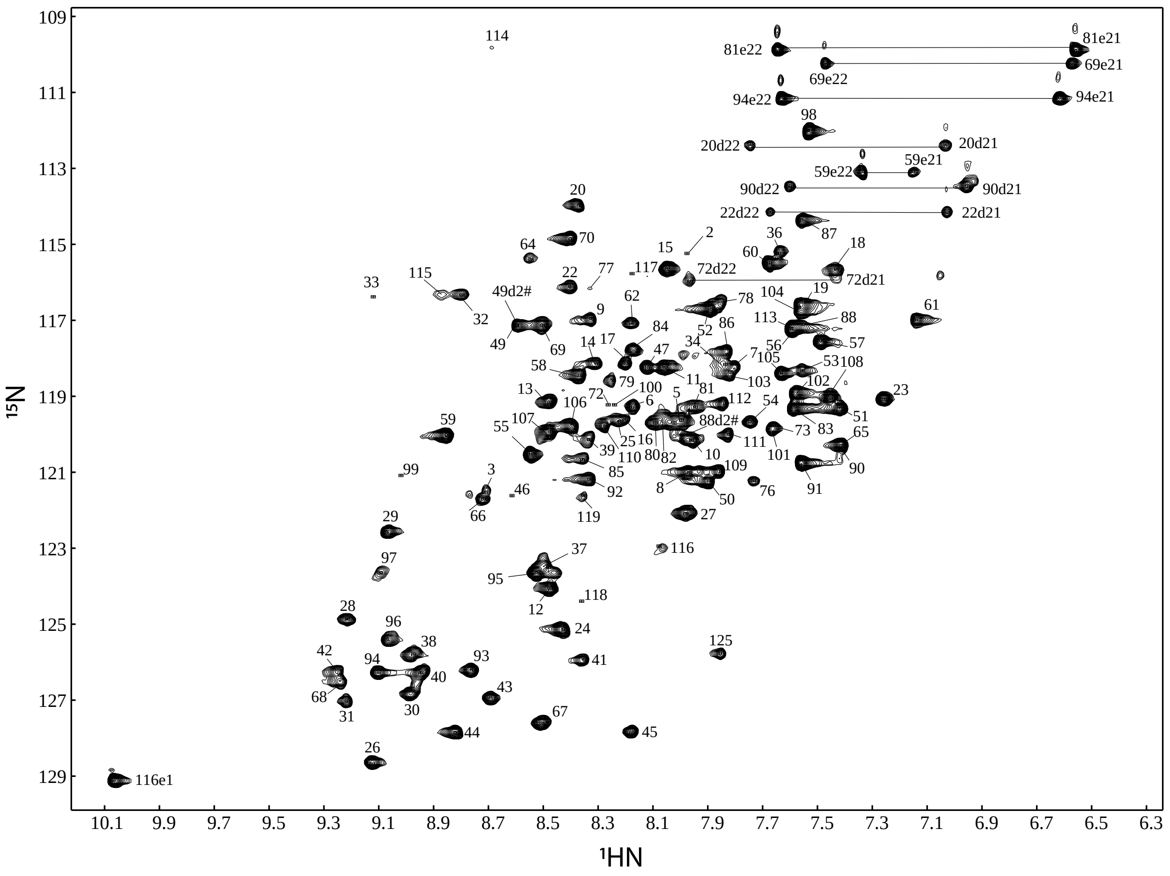

Supplementary Fig. 6 | 2D ^{1}H-^{15}N HSQC of protein NF4-04.

The HSQC spectrum was labeled in the same way as Supplementary Fig. 3.

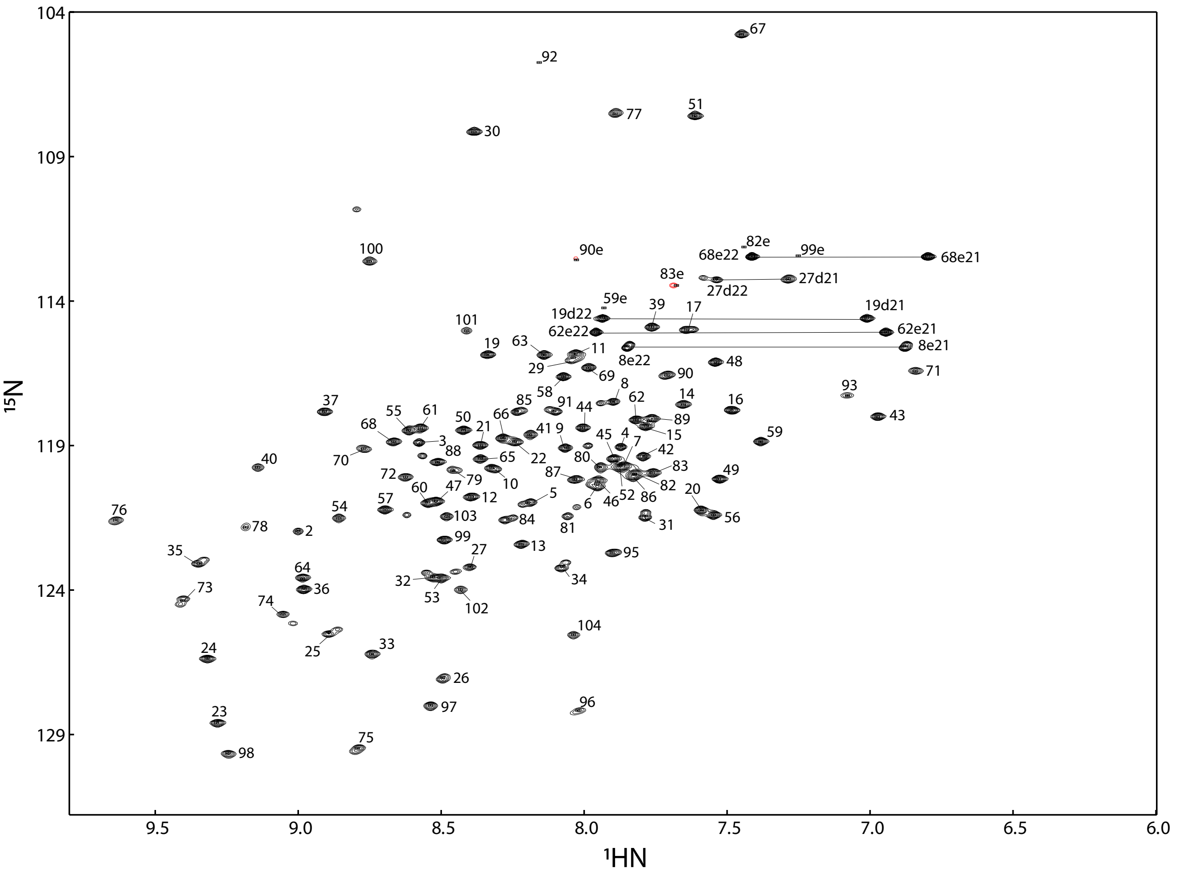

Supplementary Fig. 7 | 2D ^{1}H-^{15}N HSQC of protein NF5-03.

The HSQC spectrum was labeled in the same way as Supplementary Fig. 3.

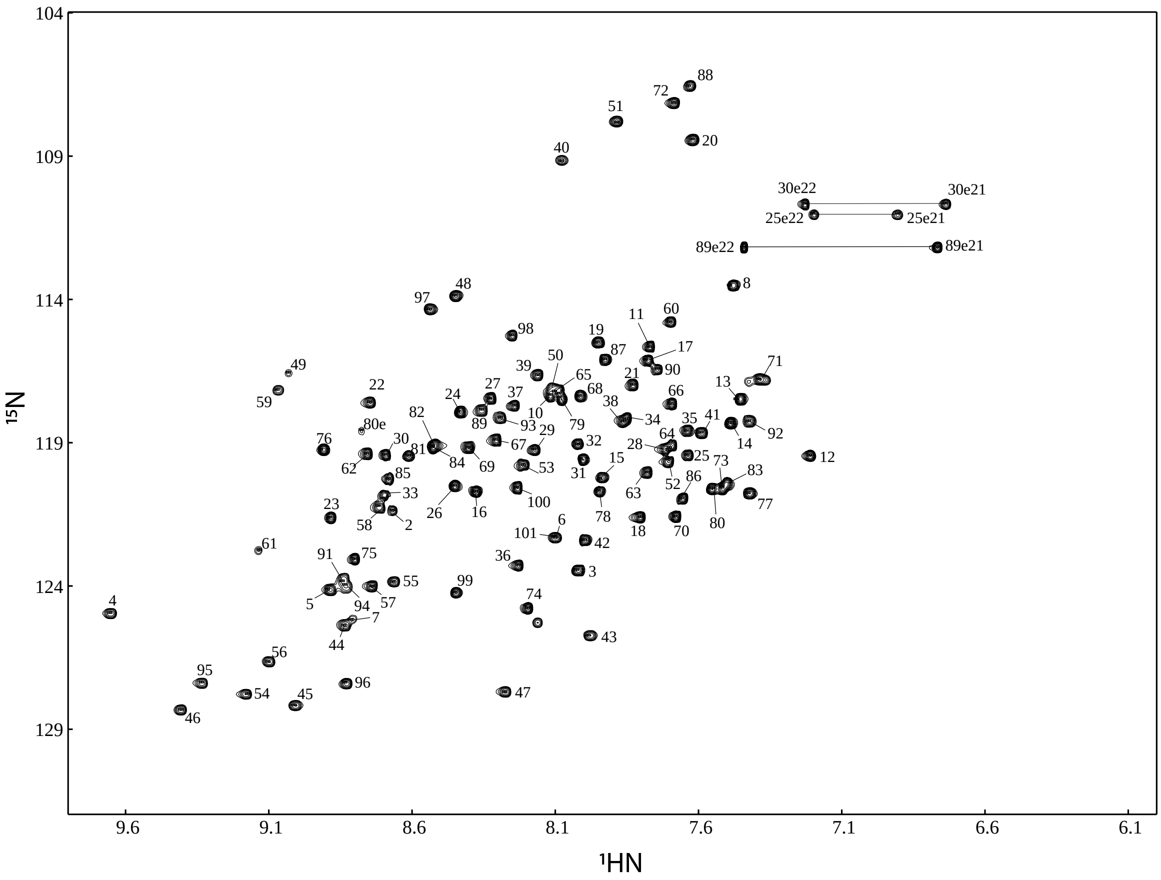

Supplementary Fig. 8 | 2D ^{1}H-^{15}N HSQC of protein NF6-02.

The HSQC spectrum was labeled in the same way as Supplementary Fig. 3.

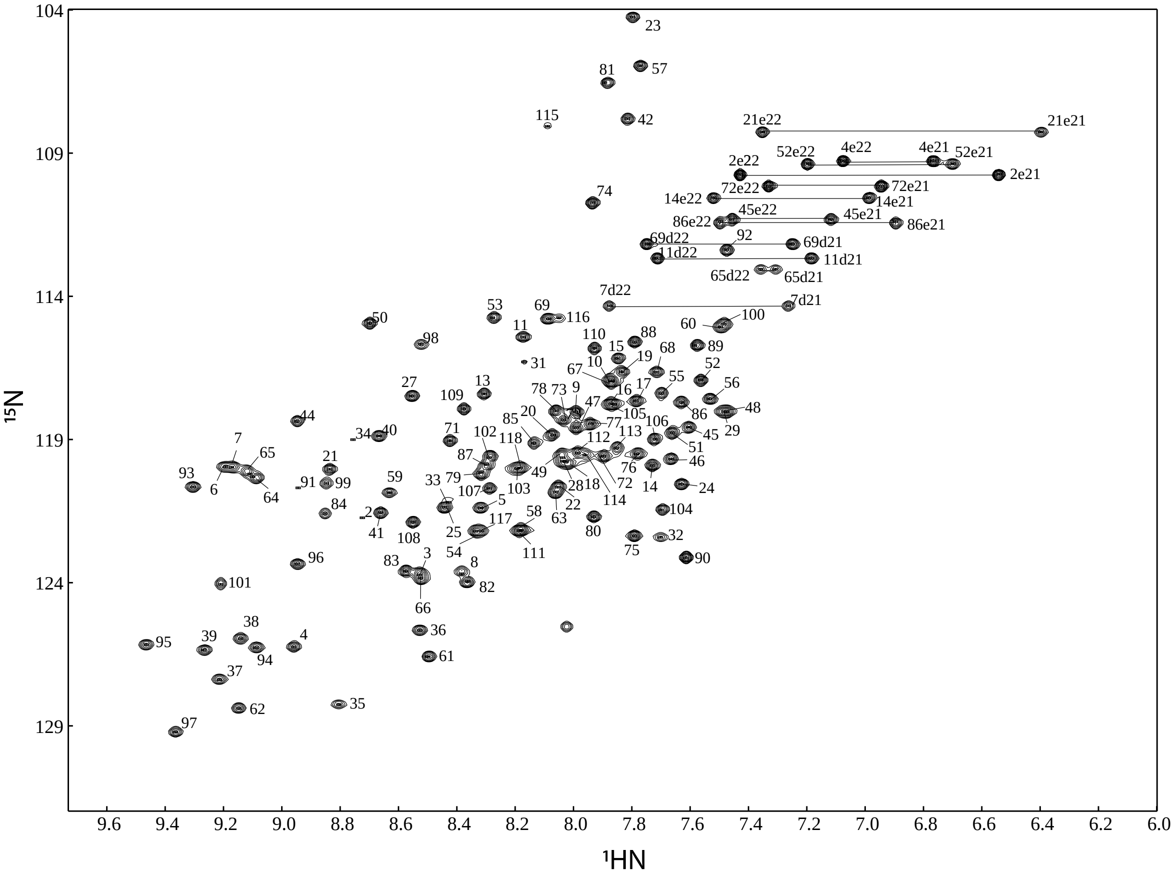

Supplementary Fig. 9 | 2D ^{1}H-^{15}N HSQC of protein NF7-04.

The HSQC spectrum was labeled in the same way as Supplementary Fig. 3.

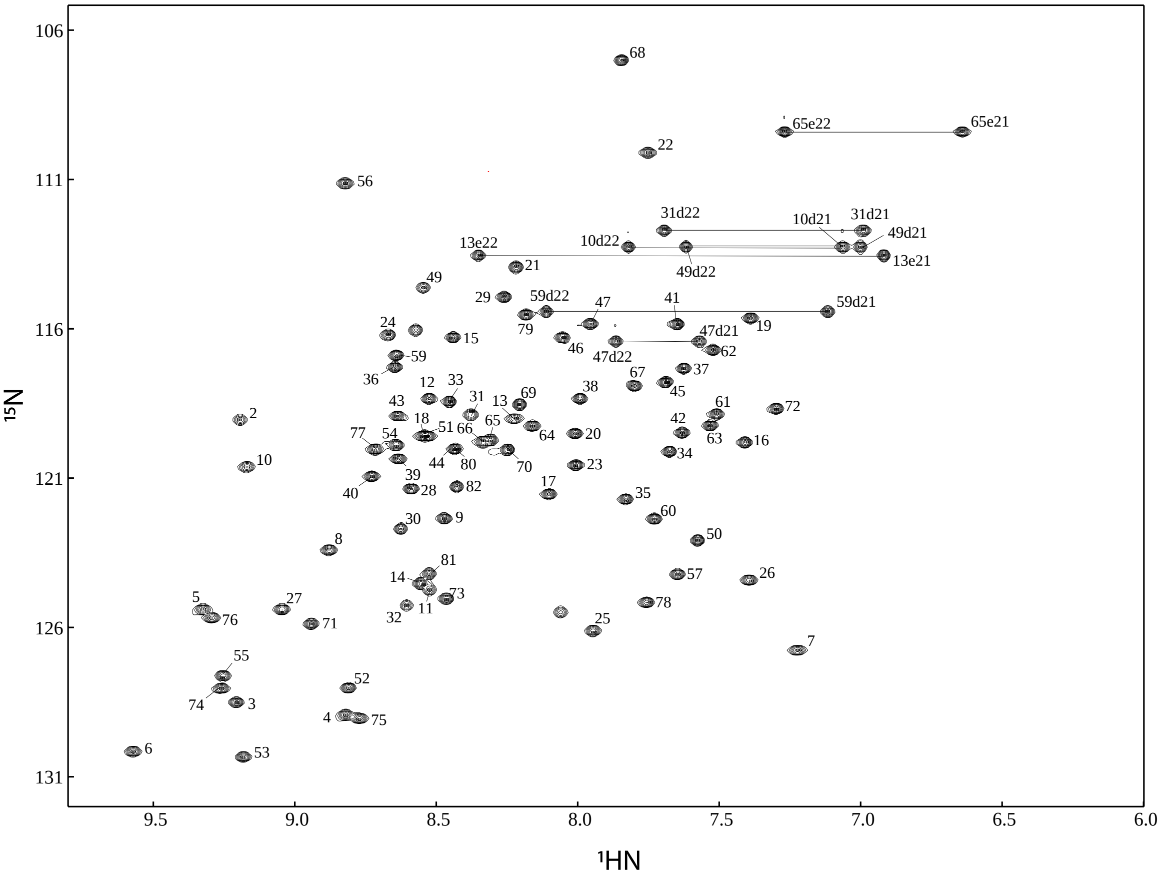

Supplementary Fig. 10 | 2D ^{1}H-^{15}N HSQC of protein NF8-01.

The HSQC spectrum was labeled in the same way as Supplementary Fig. 3.

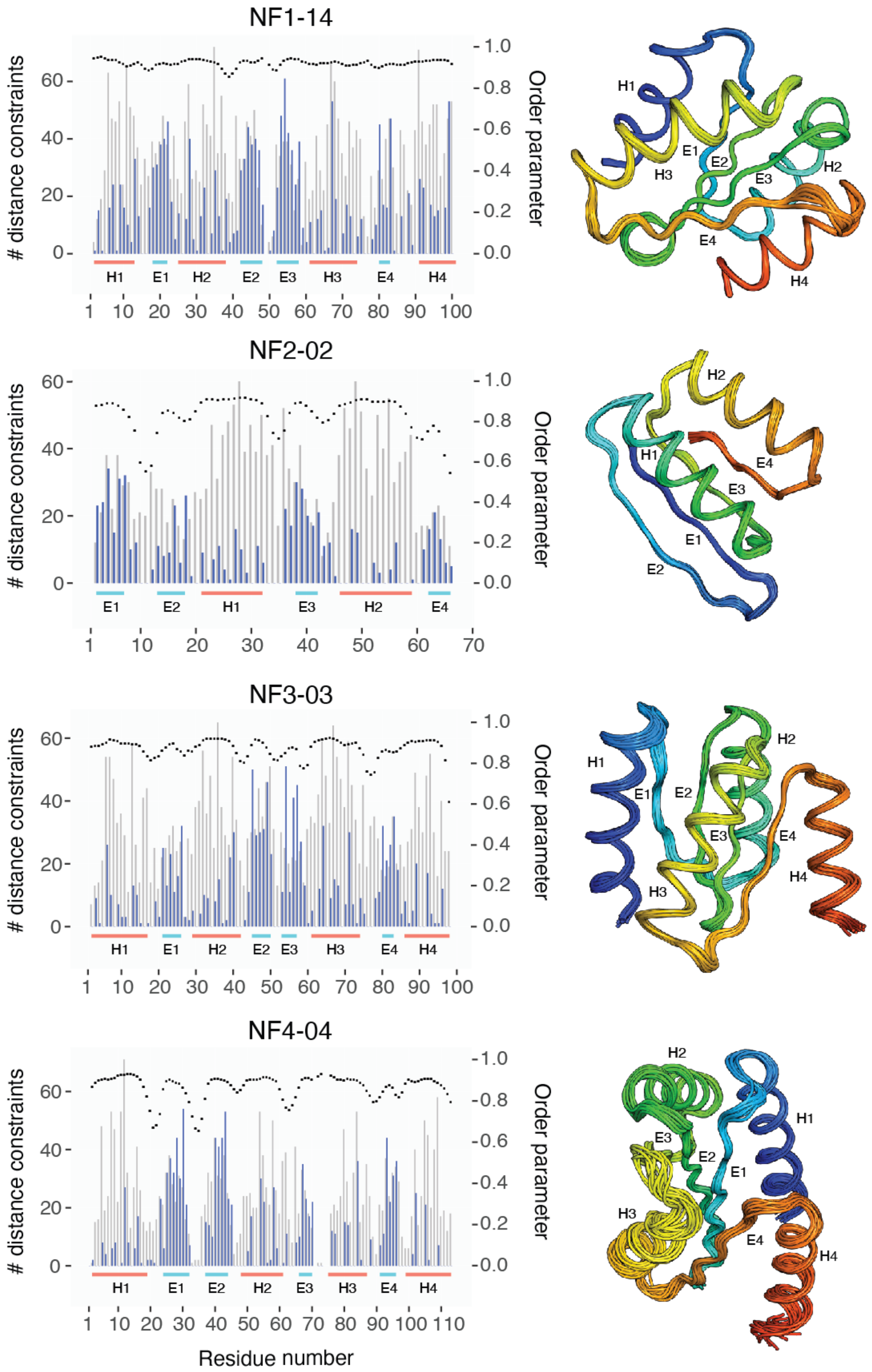
NF1-14
distance constraints
Order parameter
H1
E1
H2
E2
E3
H3
E4
H4
NF2-02
E1
E2
H1
E3
H2
E4
NF3-03
H1
E1
H2
E2
E3
H3
E4
H4
NF4-04
H1
E1
E2
H2
E3
H3
E4
H4
Residue number

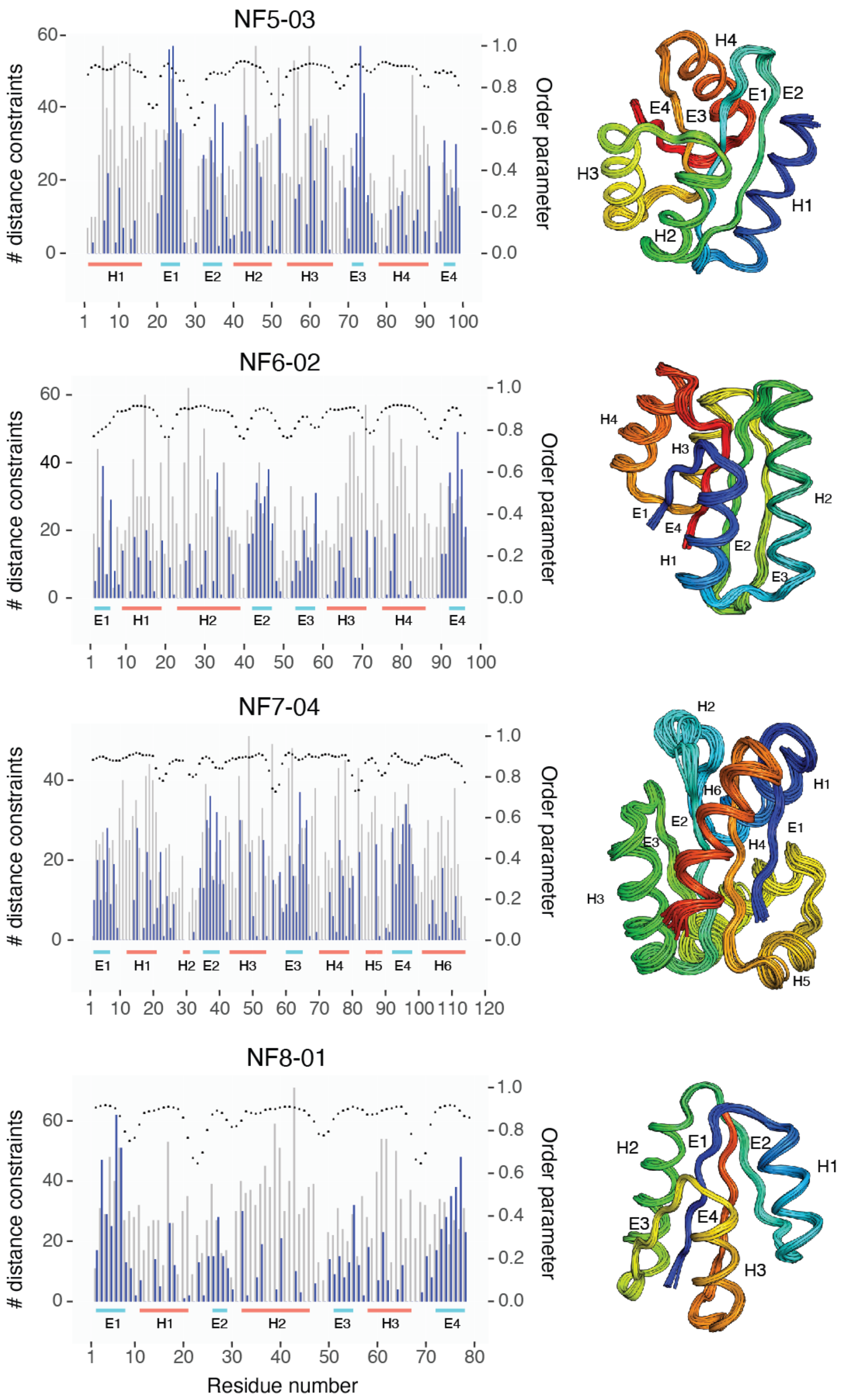
NF5-03
distance constraints
Order parameter
H1 E1 E2 H2 H3 E3 H4 E4
1 10 20 30 40 50 60 70 80 90 100
H4 E1 E2 E4 E3 H3 H1 H2
NF6-02
distance constraints
Order parameter
E1 H1 H2 E2 E3 H3 H4 E4
1 10 20 30 40 50 60 70 80 90 100
H4 H3 H2 E1 E4 E2 H1 E3
NF7-04
distance constraints
Order parameter
E1 H1 H2 E2 H3 E3 H4 H5 E4 H6
1 10 20 30 40 50 60 70 80 90 100 110 120
H2 H6 H1 E2 E1 E3 H4 H3 H5
NF8-01
distance constraints
Order parameter
E1 H1 E2 H2 E3 H3 E4
1 10 20 30 40 50 60 70 80
Residue number
H2 E1 E2 H1 E3 E4 H3

Supplementary Fig. 11 | TALOS+ order parameter and the number of NOE distance constraints for each residue.

Black dots represent the TALOS+ order parameter shown on the right vertical axis for each residue. Gray and blue bars respectively represent the number of local and non-local NOE distance constraints between the *i*-th and *j*-th residues, shown on the left vertical axis (local constraints are defined for the residues with $|i - j| < 5$, and non-local constraints are for the ones with $|i - j| \geq 5$). Red and cyan bars along the horizontal axis represent helix and strand regions defined by the DSSP calculation for the NMR models. Almost all residues show high order parameter > 0.6; most residues have local or non-local NOE distance constraints. We excluded regions with the TALOS+ order parameter less than 0.8 in the RDC validation, so the accuracy of structural determination for regions with the low order parameters cannot be guaranteed.

Supplementary Tables

ID	sequence	*E*-value
NF1-01	mGDLEKIKEAVKRVLPKAKIYEVTSEEEIERVSREIKKEGPRRILVIRSDSGSILIVISRDEKKLR<br>QMSKAAKEVSPNMTLLEFRGQDPEKIRKELERLRRGslehhhhhh	0.84
NF1-02	mGDLDKMEEAVKKVNPGAKTYKVTSPEEIKKVSEEIRKEGPRRIIVVRTDSGKILIIISRNEED<br>MRRMSEAIKRVNPNATILEFTGQDPEKIRQRLEELWRGslehhhhhh	0.14
NF1-03	mGKLEKMKRAAKKVDPRVKILEVTSEDEIKEASKRIKEEGPRRIVVVRMSQGHILIFISRDEE<br>NMRKIAKAVKKVGPEATILEFEGQDPDRIREELRKLREGswslehhhhhh	0.55
NF1-04	mGNLEKIREAVRKVLPKARTLRVNSREDIERASKEIKKEGPRRILIIRSSSGHILIVISRSEEKLE<br>RMKQAAKKVEPDATLLEFRGQDPEKIEKEMRKLYEGslehhhhhh	3.3
NF1-05	mGELEKMKRAAKKVDPRVKILEVRTPDQIEEASERIKREGPRRILVVRHDSGNILIFISRDEES<br>LKKIKEAVQRVGPRATTLEFRGQDPEKIERELRKLLKGswslehhhhhh	0.72
NF1-06	mGPLDKMAKAAKKVLPKARILRVRTPEQIERASREIKKEGPRRIIIIRADSGNILIIISRDEESAK<br>RIQEAIKRVLPNATLLEFRGQDPEKIEKELRKLLKGswslehhhhhh	0.007
NF1-07	mGDVEDIMNDAKNLSPSIQIYEVTTPDELEQAVRDIKNTGAQVVILVWTSSGKLLLFAVTNPE<br>DAERVARDAKKRNPSAKVVRLEGVPPDDIEEQARRLWKGslehhhhhh	1.2
NF1-08	mGDVDNVKKRVKEQDPNAQVYEATTPDEIEEVVKRVKKYGAQVVILVYLSDGKIIVIAVRDP<br>SVADQIIEDLKKQNPNLTIIRLEGAPPDELKRRMEELWRGslehhhhhh	0.21
NF1-09	mGTVDDIIKDAQNLSPSVQVYEVSTPDEVKEAVERVKNTGAQVVILVYTSSGKLFLFAITDPEI<br>ARKIAEDAKKRNPNAKVKRLEGKPPEDIRQQMEDLLTGslehhhhhh	0.12
NF1-10	mGSADQIIEDAQRQSPSVQVRKVSTPDEMDQAVRDVKNTGAQVVILVYTSSGELLIFAVTDPE<br>DARQIEEDAKKRNPSAKVVRLEGKPPDEIKKQMEKLLRGslehhhhhh	1.3
NF1-11	mGTADDVMQDAKDQDPNAQVYKATTPDEIREAVKRIEKTGAQVVLIIYTSSGILILVAVTNPE<br>DADKILKEAKKRNPNVTLVRLEGKPPEDIRKQAENVWKGslehhhhhh	0.65
NF1-12	mGDLDNMEKRAKELSPSVQIYRVTTPDEMEEAVQRVKNTGAQVVILVYTSSGRLIIFAVTNP<br>EIADRIKEDAKRQNPSAQLVRLEGASPDQIRQQIEDLLRGslehhhhhh	0.34
NF1-13	mGTVEEIKKDAQKLDPNAQVREVTTPDEIEEAVRQVKNYGAQVVLLFYTSSGKIVVVAVRSK<br>EVADRIAQQVKDRNPSVTVIRLEGASPEEIREQMERLWKGslehhhhhh	0.022
NF1-14	mGDADKIMEQAKRQDPNAQVYKVTTPDEIEEAVRRIEKYGAQVVLIIYTSSGIVILVAVRDPS<br>QADQILKEAKKQNPSATFVRLEGVSPDDLRRQVEDVWRGslehhhhhh	0.94
NF1-15	mGDLENIIEKAKRQNPSAQVYEATTPDELDEVAERVQRTGAQVVILVYTSSGKIIVFAVTNPE<br>DAKRIVDEAKNQNPSAKVVRLEGASEDDMKEQMRRLWKGslehhhhhh	2.1
NF1-16	mGDIENIIKDAKKQSSSVQVEKVTTPDEAEEVVRRVEKTGAQVVVLVYTSSGLVIVFAITNPEI<br>AKRIVQRAKEQNPSATVKRLEGVSPDEIQEEIERLLKGslehhhhhh	0.17

Supplementary Table 1 | Designed sequences of the series of NF1.

Computationally designed sequences are shown in uppercase and residues added to allow expression, purification, and the spacer between the designed sequence and the C-terminal 6xHis-tag are shown in lowercase. '*E*-value' column shows the smallest *E*-value obtained from a PSI-BLAST search against nr database.

ID	sequence	E-value
NF2-01	mgSEIRLESSDGQDKTYTATSDDELKEILERAVKEGIKRIEIRGASERMLRTSEEIARRAGIEW RKNGslehhhhhh	2.8
NF2-02	mgTEIELESKNGQREHYTATSEDEARKIIEKAVRRGIKRIELRGASEQLIRDMQEIAKQIGLQY RTDGslehhhhhh	4.2
NF2-03	mgTKIHAEGPNGETRTYTATSEEEAEKIIRELVKRGIQRIELQGASEDLLRKMEEIARRAGIEY RTDGslehhhhhh	0.015
NF2-04	mgKTIEAESSDGETRTYTATSEEEAERIIRKLQKEGIQRIRLQGVSEDLRKRLEELARKIGLQW RYRKgslehhhhhh	0.16

Supplementary Table 2 | Designed sequences of the series of NF2.

The designed sequences were given in the same way as Supplementary Table 1.

ID	sequence	E-value
NF3-01	mGDDDSLRKRLEEDAKKSGKRVEFRRYNDPKRIEEELRRARKDGETLVILVGGVIVIVSNDE KLVREIKKNLQKERPDKETISVTTEEDIKRALKRIKEGswslehhhhhh	0.32
NF3-02	mGSSEKIRKKLEELAKRTGKRVQFREYNDTEQVRKTLEEAQRRGETLVVLSRGTVIIVSTNEE LIRRIEELVKQSNPNLETYEATTDEDIERILRELDKGslehhhhhh	0.34
NF3-03	mGSDEEIRKKLEELAKRKGKDLQLRRYNDPNEVEKSIREALKKGRTLIIIINGVFVVVSTDEDL IREIKRLIKESNPNKKTLDVTTEEDLEEVLRRIKKGswslehhhhhh	0.001
NF3-04	mGDSERLERRIRERAKKTGKDLQFREYDSPDKVRESLRKAQEKGRTLIVLVRGTIIVVSPDPE LARQIVEDLQKERPDLTTLEATTEDDIRKQLKRLREGswslehhhhhh	0.52

Supplementary Table 3 | Designed sequences of the series of NF3.

The designed sequences were given in the same way as Supplementary Table 1.

ID	sequence	E-value
NF4-01	mGSEEIYRLIEKIWRDIKNENPNARILIFIVFTSDGKIEVIIVIIDDDEELLRRIEETAKKRVPKV EIRRDRNEERAQKKIEEELKKRNPNATLYTVTSLDELEEILKKLTKQEGslehhhhhh	4.2
NF4-02	mGSRKVYELVRKVWEAIKEENPNAKILIFLLFTSDGTIQIIIVIIDSSEETLRRIEEQIRKRVPNV RIERSKNEEEARKEIEKELKDRDPNATLYEVTTKEELKKILEKLERQEGslehhhhhh	0.14
NF4-03	mGSERIYKIIKEIWETIKKENPKVKILIFILLTSDGTIEIIIVVISSNREEAQRIVEELQKRYPEVEI RRSENEEQASREIKQELKDRNPTATIYEVTSPEELTKILEKLKKQEGslehhhhhh	0.88
NF4-04	mGSEEIRELVRKIYETVRKENPNVKILIFIIFTSDGTIKVIIVIIADDPNDAKRIVKKIQERFPKLT IKQSRNEEEAEKRIQKELEERNPNAEIQVVRSEDELKEILDKLDEKKGswslehhhhhh	0.52
NF4-05	mGEDELKEQITRVWRTIKKENPGVKILVFILFSSNGEIQVIIVIISDNEDELRELEERAKRRVPK VEIRRDRDEQKASDKIREELKRRDPNAEIFEVTSEEELKKIIEELKRQKGslehhhhhh	6.6
NF4-06	mGTDKVEELVRKIYESVEKENPNAKILIFLVYTSDGKILIIIVIISSSEETAKKIVEELKRRFPEV EIRQDRDEESAKKKIEEELRRRDPNATIYKVTSKDELEKILEKLRRDEGslehhhhhh	0.18

Supplementary Table 4 | Designed sequences of the series of NF4.

The designed sequences were given in the same way as Supplementary Table 1.

ID	sequence	E-value
NF5-01	mGEEERVKREAKRIEDEDPNRKILIYIDSNGEIEIKEVTSPEDVRKILEKLGVSEDLLREIER AVKNGEYDLFFIVKTEESTRRAEEIRERLGKPVRIETGslehhhhhh	0.44
NF5-02	mGEEEKILRKADKIIKEDPNRKIIIIINPDGKIELREVTSEEDVREILERMGVPPDLLKEIERA VRNGEYDLFFIVTTEESERRARKLKKQMGKPVLIETGswslehhhhhh	0.039
NF5-03	mGEDDEILQRAKDILKEDPNRKILIILNPDGKIELYEVTSEEDIKRIAKKAGISEELLRRILQS FRDGQYDLFFIAKTEDDERRARELKERMGKPVEILRGslehhhhhh	0.36
NF5-04	mGDKEKVEETFRKIEEEDPNRKIIIIINPDGKIEIRTVTSPEDLERIFRKMGISEDLLRTAKE SLREGQYDLFFITKTEESRRTAEELKKRLGKPVLILTGswslehhhhhh	0.2
NF5-05	mGNDEKVKETARKLLEKNPKVKIIIILNPDGEIRVKTVTSPDDLEQIARESGLPPDLLEQAL RDLKNGQYDLFVFAKTEEDERRARQLKEDMGKPVLILRGswslehhhhhh	2.3
NF5-06	mGNEDEARERIRKIEDEDPNAKIIIYLTPDGELRVQKVTSEDDVEEFLKKMGVPPELLKRI LESLRNGEYDLFFIVRTEESEKRARKLKERLGKPVEILRGslehhhhhh	1.1

Supplementary Table 5 | Designed sequences of the series of NF5.

The designed sequences were given in the same way as Supplementary Table 1.

ID	sequence	E-value
NF6-01	mGELYTVDSPDEVRRIAKELGLSEEQLRRIEKEFRRAEKKGKTVLVYIDSNGEVRIREVTSE DELRELLQRLGVDPEIIERIERKFNNGEIKLVIIKGslehhhhhh	0.11
NF6-02	mGKLYEVDSPDSVEKIARELGLSEEQLRRIQKEFERAERKGKLVIVYLTSDGKVEIREVTSE EELEKILKKLGVDEEIIRRIKRLRKEGQIKLVIIEGslehhhhhh	0.002
NF6-03	mGELRTVDSPDSVREIIKRLGLSEEQLRRIEKKFKELEKKGRTLLVYIDSNGNVELRTVTSE DELERILRELGVDEEILRRVKELFREGQVKLVIIIGswslehhhhhh	0.15
NF6-04	mGELRTVTSEDEVEKIARQLGLSEEQLRRILKKFREVEKRGKLLIVYLTSDGEVRIQEVTTK DELSRILKELGVDPEIRERIRKEFEEGQVKLVFIKGswslehhhhhh	0.1
NF6-05	mGELRTVTTKEDVRRFAKKTGLSEDQLRKIEERFEEAEKKGKTIIVYITSDGKVEIQEVDTE DELKRILNELGVDPDIKEKIRKKFENGEVKLVFILGswslehhhhhh	0.24
NF6-06	mGKLYRVDSPDSVRKIAKELGLSEEQLRRIEKSFKEVEKKGKTLIVWLDSNGNVEEQTVDT KDELSRILNELGVDPEIKERIRKLFENGEVKLVIIEGslehhhhhh	0.26

Supplementary Table 6 | Designed sequences of the series of NF6.

The designed sequences were given in the same way as Supplementary Table 1.

ID	sequence	E-value
NF7-01	mGLVQRFDVDENSEQVERLIRIAGLDEDKFEKAEIIVIVIVKTEEKLKRLAQRVKDLGADIILE INMDENSETVKRLAKEAGIPPDELRRAEILILVLVKTEEKAQRISEQIKRQGswslehhhhhh	0.15
NF7-02	mGETTQFDVDENSEKVKRLIRKAGLSEEELKKADIIVIVISRNPEELKRLEEIVRNLGADRIIK LNVDENPEQVRQFAEEAGIPPEKLKRIDYLVVIISKTKEEAKELAERIKRQGswslehhhhhh	0.27
NF7-03	mGTTRQYDVDENPETVEKLIRIAGIPKEELDRADIIVFILSRSEEKARRLKKKIQSLGADRIQII NVDENSEEVKRFAKTAGISEEELRKSEYLIIVISKTKDEAKRLSEEIKKQGslehhhhhh	0.48
NF7-04	mGQIQYFNVDENPEQVRKLIEQAGLDPDELREAEVIIIIISRTPEQLEKLSRQVKELGADRLLE FNVDENPEQASKLAKTAGISEKQLREADYIILILVRDEKKAKKFADSLRKKGslehhhhhh	0.51
NF7-05	mGDVQRFDVDENSEQVERLARQAGLSEDELRKAKIIIILISRTEKKLRELEEQVKRLGADRWI LLNVDENPEQVEKLIRTAGISEDEFKRSEIIILILSKTKDEAEELSRRLKKTGslehhhhhh	2.3
NF7-06	mGTVRRYDVDENSDQVKNLIKIAGLDPEKLKRSEIIVIIVVKTPEKLKRLSQQVKDLGADIIIEI NVDENSEQVQRLADEAGIPPEDLKKAEYLIFVLSKTKDKAEQISRDLEKKGslehhhhhh	0.012

Supplementary Table 7 | Designed sequences of the series of NF7.

The designed sequences were given in the same way as Supplementary Table 1.

ID	sequence	E-value
NF8-01	mGTILIFLDKNKEQAEKLAKEVGVTEIYESDNLEELYREIKERIERENPNATILTVTDPNELKKIQDEGKVDRIILLIKGslehhhhhh	1.3
NF8-02	mGTIVIFLVENEERARRIAKELGATEIYKSDNLEEAERQISKELKKENPNAEILTVTDPEEVRRRREEGQLDRLIVIIKGswslehhhhhh	3.1
NF8-03	mGTYLIFLTDDKKTAEELARKLGVTEIYESDNLEELLRRIQEEIKKRNPNAEILTVTNPDEVRRIKEEGKVDRLILIIRGslehhhhhh	0.57
NF8-04	mGTILIFFSKSEETAERIAKELGATEIYKSDNEEEALRRLSKEIKKRNPNAEIRYTTNEDEVRKRQKNGQIDRLIIVILGslehhhhhh	1.4
NF8-05	mGTILIFFSKNPEEIERLAKELGATRIYESDNEEEAYRRLEEQIRRENPNATVLRVTSPSEIRQKKKEGKVDLLIFVLIGslehhhhhh	0.62
NF8-06	mGTILIFFTKDEETLRKLAKEVGVTEIYESDNEDEAYKRLKEEIRKRNPNAEILRVTDENELRRLRKEGQVDQFILVLIGslehhhhhh	0.22
NF8-07	mGTIILFFTTDPNEAKKVYKELGATRITRSDNEEEASRRLKEEIERRNPNAKIYRYTTPEEARRTAEKEGATEIIIILIGslehhhhhh	2.0
NF8-08	mGTIVIIFVTDPKEAEDIYQQLGATRITTSDNEDEARKELSRELKEENPNAKIYSYTTEDEAERTAREQGVTRIIIVLVGslehhhhhh	1.5
NF8-09	mGTIIIFFTTNPEEAEKVYKKLGATEITTSRNEDEARERLEEKIRRENPNAKIYSTTNPDDAEKTAKNQGATRIIIVLIGslehhhhhh	0.13
NF8-10	mGTIIIIFSTNPEDVKTIYKKLGVTRLTRSDNEDEARKRLSEEIKRENPNAKIISTTTPDEAKREQEQQGATRIILVEIGswslehhhhhh	0.57
NF8-11	mGTIIIIIVTDPNEAKTIYKELGATEIYESDNEEELLRRIEEQLRRENPNAKIYTTKNKEEAERTAREQGATRIIILLVGslehhhhhh	1.1
NF8-12	mGTIIIIFVTDPNEAQRIYDELGATRIYKSRNEDEAREELRREIEEENPNATIISTKSEKEARKQQETQGATEIIIVLIGslehhhhhh	2.5

Supplementary Table 8 | Designed sequences of the series of NF8.

The designed sequences were given in the same way as Supplementary Table 1.

	RMSD between design and NMR (Å)	
	**Backbone (N, Cα, C', O)**	**All heavy atoms**
**NF1-14**	1.4	2.0
**NF2-02**	1.5	1.9
**NF3-03**	2.0	2.4
**NF4-04**	1.9	3.1
**NF5-03**	1.9	2.6
**NF6-02**	1.5	2.2
**NF7-04**	1.7	2.3
**NF8-01**	1.4	2.0

Supplementary Table 9 | RMSD between the design model and NMR structure.

The average RMSD between the design model and the 20 NMR structures using backbone heavy atoms and all heavy atoms are shown.

Design ID	Expressed	Soluble	αβ-protein CD spectrum (20 °C)	Monomeric	Well-resolved HSQC
NF1-01	Y	Y	Y	N	
NF1-02	Y	Y	Y	Y	Y
NF1-03	Y	Y	Y	N	
NF1-04	Y	Y	Y	N	
NF1-05	Y	N			
NF1-06	Y	N			
NF1-07	Y	Y	Y*	N	
NF1-08	Y	Y	Y	Y	N
NF1-09	Y	Y	Y*	N	
NF1-10	Y	Y	Y	Y	Y
NF1-11	Y	Y	Y*	N	
NF1-12	Y	Y	Y	N	
NF1-13	Y	Y	Y	Y	Y
NF1-14	Y	Y	Y	Y	Y
NF1-15	Y	Y	Y	Y	N
NF1-16	Y	Y	Y*	N	

Supplementary Table 10 | Experimental summary of a series of designs for NF1.

Each row corresponds to the results for each design. The columns give the results for each experimental characterization, of which the summaries are described in Extended Data Table 1. Each characterization was performed sequentially from the left to the right; well-behaved designs at a characterization (Y) are then evaluated by the next one and not well-behaved designs (N) end being evaluated.

* The CD spectrum was characteristic of αβ-proteins, but looked partially unfolded.

Design ID	Expressed	Soluble	αβ-protein CD spectrum (20 °C)	Monomeric	Well-resolved HSQC
NF2-01	Y	Y	Y	Y	N
NF2-02	Y	Y	Y	Y	Y
NF2-03	Y	Y	Y	Y	Y
NF2-04	Y	Y	Y	Y	N

Supplementary Table 11 | Experimental summary of a series of designs for NF2.

The summary was given in the same way as Supplementary Table 10.

Design ID	Expressed	Soluble	αβ-protein CD spectrum (20 °C)	Monomeric	Well-resolved HSQC
NF3-01	Y	Y	Y	Y	Y
NF3-02	Y	N			
NF3-03	Y	Y	Y	Y	Y
NF3-04	Y	Y	Y	Y	†

Supplementary Table 12 | Experimental summary of a series of designs for NF3.

The summary was given in the same way as Supplementary Table 10.

† The HSQC measurement was not conducted due to low concentration.

Design ID	Expressed	Soluble	αβ-protein CD spectrum (20 °C)	Monomeric	Well-resolved HSQC
NF4-01	Y	Y	Y	N	
NF4-02	Y	Y	Y	Y	‡
NF4-03	Y	Y	Y	Y	Y
NF4-04	Y	Y	Y	Y	Y
NF4-05	Y	Y	Y	Y	Y
NF4-06	Y	Y	Y	Y	Y

Supplementary Table 13 | Experimental summary of a series of designs for NF4.

The summary was given in the same way as Supplementary Table 10.

‡ The HSQC measurement was not conducted due to not small amount of dimeric state (the second peak of SEC-MALS).

Design ID	Expressed	Soluble	αβ-protein CD spectrum (20 °C)	Monomeric	Well-resolved HSQC
NF5-01	Y	Y	Y*	N	
NF5-02	Y	Y	Y*	N	
NF5-03	Y	Y	Y	Y	Y
NF5-04	Y	Y	Y	N	
NF5-05	Y	Y	Y	N	
NF5-06	Y	Y	Y	Y	Y

Supplementary Table 14 | Experimental summary of a series of designs for NF5.

The summary was given in the same way as Supplementary Table 10.

* The CD spectrum was characteristic of αβ-proteins, but looked partially unfolded.

Design ID	Expressed	Soluble	αβ-protein CD spectrum (20 °C)	Monomeric	Well-resolved HSQC
NF6-01	Y	Y	Y	Y	Y
NF6-02	Y	Y	Y	Y	Y
NF6-03	Y	Y	Y	N	
NF6-04	Y	Y	Y	Y	Y
NF6-05	Y	Y	Y	Y	Y
NF6-06	Y	Y	Y	Y	Y

Supplementary Table 15 | Experimental summary of a series of designs for NF6.

The summary was given in the same way as Supplementary Table 10.

Design ID	Expressed	Soluble	αβ-protein CD spectrum (20 °C)	Monomeric	Well-resolved HSQC
NF7-01	Y	Y	Y*	N	
NF7-02	Y	Y	Y	Y	Y
NF7-03	Y	Y	Y	Y	Y
NF7-04	Y	Y	Y	Y	Y
NF7-05	Y	Y	Y*	N	
NF7-06	Y	Y	Y*	N	

Supplementary Table 16 | Experimental summary of a series of designs for NF7.

The summary was given in the same way as Supplementary Table 10.

* The CD spectrum was characteristic of αβ-proteins, but looked partially unfolded.

Design ID	Backbone type	Expressed	Soluble	αβ-protein CD spectrum (20 °C)	Monomeric	Well-resolved HSQC
NF8-01	1	Y	Y	Y	Y	Y
NF8-02	1	Y	Y	Y	Y	Y
NF8-03	1	Y	Y	Y	N	
NF8-04	1	Y	Y	Y	Y	Y
NF8-05	1	Y	Y	Y	Y	Y
NF8-06	1	Y	Y	Y	Y	Y
NF8-07	2	Y	N			
NF8-08	2	Y	Y	Y*	N	
NF8-09	2	Y	Y	Y	N	
NF8-10	2	Y	Y	Y	N	
NF8-11	2	Y	Y	Y	Y	Y
NF8-12	2	Y	Y	Y	N	

Supplementary Table 17 | Experimental summary of a series of designs for NF8.

The summary was given in the same way as Supplementary Table 10. The backbone type 1 has GABA loop immediately before the last strand and 2 has GBA loop (see Extended Data Fig. 6 for details).

* The CD spectrum was characteristic of αβ-proteins, but looked partially unfolded.

References

1. Zweckstetter, M. & Bax, A. Prediction of sterically induced alignment in a dilute liquid crystalline phase: Aid to protein structure determination by NMR. *Journal of the American Chemical Society* **122**, 3791-3792 (2000).
2. Cheng, H. et al. ECOD: an evolutionary classification of protein domains. *PLoS Comput Biol* **10**, e1003926 (2014).
