# Supplementary material for: Exploration of novel αβ-protein folds through de novo design
Source: Nat Struct Mol Biol. 2023 Jul 3;30(8):1132–40. doi: 10.1038/s41594-023-01029-0 (PMC10442233; doi:10.1038/s41594-023-01029-0)
Supplement: Supplementary file 2 — Reporting Summary [file 41594_2023_1029_MOESM2_ESM.pdf]

nature portfolio

Corresponding author(s): Nobuyasu Koga

Last updated by author(s): 2023/3/17

Reporting Summary

Nature Portfolio wishes to improve the reproducibility of the work that we publish. This form provides structure for consistency and transparency in reporting. For further information on Nature Portfolio policies, see our Editorial Policies and the Editorial Policy Checklist.

Statistics

For all statistical analyses, confirm that the following items are present in the figure legend, table legend, main text, or Methods section.

n/a	Confirmed	
☐	☒	The exact sample size (*n*) for each experimental group/condition, given as a discrete number and unit of measurement
☐	☒	A statement on whether measurements were taken from distinct samples or whether the same sample was measured repeatedly
☒	☐	The statistical test(s) used AND whether they are one- or two-sided<br>*Only common tests should be described solely by name; describe more complex techniques in the Methods section.*
☒	☐	A description of all covariates tested
☒	☐	A description of any assumptions or corrections, such as tests of normality and adjustment for multiple comparisons
☐	☒	A full description of the statistical parameters including central tendency (e.g. means) or other basic estimates (e.g. regression coefficient) AND variation (e.g. standard deviation) or associated estimates of uncertainty (e.g. confidence intervals)
☒	☐	For null hypothesis testing, the test statistic (e.g. *F*, *t*, *r*) with confidence intervals, effect sizes, degrees of freedom and *P* value noted<br>*Give P values as exact values whenever suitable.*
☒	☐	For Bayesian analysis, information on the choice of priors and Markov chain Monte Carlo settings
☒	☐	For hierarchical and complex designs, identification of the appropriate level for tests and full reporting of outcomes
☒	☐	Estimates of effect sizes (e.g. Cohen's *d*, Pearson's *r*), indicating how they were calculated

Our web collection on statistics for biologists contains articles on many of the points above.

Software and code

Policy information about availability of computer code

Data collection	Rosetta software suite 3 was used for protein design and folding calculations.<br>JASCO SpectraManager software v2 was used for CD.
Data analysis	The codes for creating a list of all β-sheet topologies, calculating frustrations for a given β-sheet topology, calculating β-sheet topology for a given pdb file, and calculating occupancy ratio for each ECOD domain entry will be available at https://github.com/kogalab23/novel_ab-fold_design.<br>The database analysis for the investigation of similar naturally occurring protein structures was performed by MICAN (2018.04.05) and TM-align (20120126).<br>For all obtained domain structures from ECOD database (20180423-(develop210)-F99), structure refinements were performed using ModRefiner, and the secondary structures were assigned using STRIDE.<br>SEC-MALS data were analyzed by ASTRA software 6.1.2.<br>HSQC data were analyzed by Delta 5.0.4 NMR software.<br>All NMR structure analyses were done as described in the methods section with the following programs: MagRO-NMRViewJ (updated version of Kujira),Filt_Robot, TALOS+ 2017, FLYA 3.98.5, CYANA 3.98.5, Amber12, and PALES 2.1.

For manuscripts utilizing custom algorithms or software that are central to the research but not yet described in published literature, software must be made available to editors and reviewers. We strongly encourage code deposition in a community repository (e.g. GitHub). See the Nature Portfolio guidelines for submitting code & software for further information.

Data

Policy information about availability of data

All manuscripts must include a data availability statement. This statement should provide the following information, where applicable:

- Accession codes, unique identifiers, or web links for publicly available datasets
- A description of any restrictions on data availability
- For clinical datasets or third party data, please ensure that the statement adheres to our policy

The solution NMR structures have been deposited in the wwPDB as PDB 7BPL (NF1-14), 7BPM (NF2-02), 7BQE (NF3-03), 7BQC (NF4-04), 7BPP (NF5-03), 7BQB (NF6-02), 7BPN (NF7-04), and 7BQD (NF8-01). The NMR data have been deposited in the BMRB under the accession numbers 36327 (NF1-14), 36328 (NF2-02), 36334 (NF3-03), 36332 (NF4-04), 36330 (NF5-03), 36331 (NF6-02), 36329 (NF7-04), and 36333 (NF8-01). The computational design models are presented as Supplementary Data 1. The plasmids encoding the designed sequences are available from the author upon request. ECOD database is available at http://prodata.swmed.edu/ecod. A dataset obtained from cullpdb database will be available at https://github.com/kogalab23/novel_ab-fold_design.

Field-specific reporting

Please select the one below that is the best fit for your research. If you are not sure, read the appropriate sections before making your selection.

☒ Life sciences ☐ Behavioural & social sciences ☐ Ecological, evolutionary & environmental sciences

For a reference copy of the document with all sections, see nature.com/documents/nr-reporting-summary-flat.pdf

Life sciences study design

All studies must disclose on these points even when the disclosure is negative.

Sample size	Computational designs that passed selection criteria were experimentally tested. Based on the previously reported success rate of de novo designed proteins of similar size (N. Koga et al. Nature, 2012; Y.-R. Lin et al., PNAS, 2015), we estimated the number of designs we should test in order to be successful.
Data exclusions	No data were excluded
Replication	The representative designs for NMR structure determination were purified, verified by SDS-PAGE, mass spectrometry, and HSQC measurements more than once. All attempts at replication were successful.
Randomization	Randomization is not relevant to our study. This is an observational study, which does not involve evaluation of conditional effects.
Blinding	Blinding is not relevant to our study. Keeping track of the identity of each designed protein was necessary for characterizing biophysical properties and solving the structure.

Reporting for specific materials, systems and methods

We require information from authors about some types of materials, experimental systems and methods used in many studies. Here, indicate whether each material, system or method listed is relevant to your study. If you are not sure if a list item applies to your research, read the appropriate section before selecting a response.

Materials & experimental systems

n/a	Involved in the study	
☒	☐	Antibodies
☒	☐	Eukaryotic cell lines
☒	☐	Palaeontology and archaeology
☒	☐	Animals and other organisms
☒	☐	Human research participants
☒	☐	Clinical data
☒	☐	Dual use research of concern

Methods

n/a	Involved in the study	
☒	☐	ChIP-seq
☒	☐	Flow cytometry
☒	☐	MRI-based neuroimaging
